# Urinary excretion of asymmetric (ADMA) and symmetric (SDMA) dimethylarginine is positively related to nitric oxide level in tissues of normotensive and hypertensive rats

**DOI:** 10.1007/s00726-023-03246-9

**Published:** 2023-02-19

**Authors:** Dominika Szlęzak, Marcin Ufnal, Adrian Drapała, Emilia Samborowska, Maria Wróbel

**Affiliations:** 1 Chair of Medical Biochemistry, Faculty of Medicine, Jagiellonian University Medical College, 7 Kopernika St, 31-034 Kraków, Poland; 2 Department of Experimental Physiology and Pathophysiology, Laboratory of the Centre for Preclinical Research, Medical University of Warsaw, 1B Banacha St, 02-097 Warsaw, Poland; 3 Mass Spectrometry Laboratory, Institute of Biochemistry and Biophysics, Polish Academy of Sciences, 5a Pawińskiego St, 02-106 Warsaw, Poland

**Keywords:** Nitric oxide, Asymmetric dimethylarginine (ADMA), Symmetric dimethylarginine (SDMA), Aging, Hypertension

## Abstract

Nitric oxide (NO) is one of the gaseous transmitters which play a very important role in the regulation of the circulatory system. Decreased NO availability is associated with hypertension, cardiovascular and kidney diseases. Endogenous NO is generated enzymatically by nitric oxide synthase (NOS) depending on the availability of the substrate, cofactors, or presence/absence of inhibitors, such as asymmetric dimethylarginine (ADMA) and symmetric dimethylarginine (SDMA). The objective of this study was to evaluate the potential relationship between NO level in rat tissues (heart and kidneys) and the concentrations of endogenous metabolites related to NO in plasma and urine. The experiment was carried out with 16- and 60-week-old male Wistar Kyoto (WKY) and age-matched male Spontaneously Hypertensive Rats (SHR). NO level in tissue homogenates was determined by the colorimetric method. RT-qPCR was used to verify the expression of the eNOS (endothelial NOS) gene. Plasma and urine concentrations of arginine, ornithine, citrulline, and dimethylarginines were examined by the UPLC-MS/MS method. 16-week-old WKY rats had the highest tissue NO and plasma citrulline levels. Furthermore, 16-week-old WKY rats showed higher urinary excretion of ADMA/SDMA compared to other experimental groups, however, plasma concentrations of arginine, ADMA, and SDMA were comparable between the groups. In conclusion, our research shows that hypertension and aging decrease tissue NO levels and are associated with reduced urinary excretion of NOS inhibitors, i.e., ADMA and SDMA.

## Introduction

**Fig. 1:**
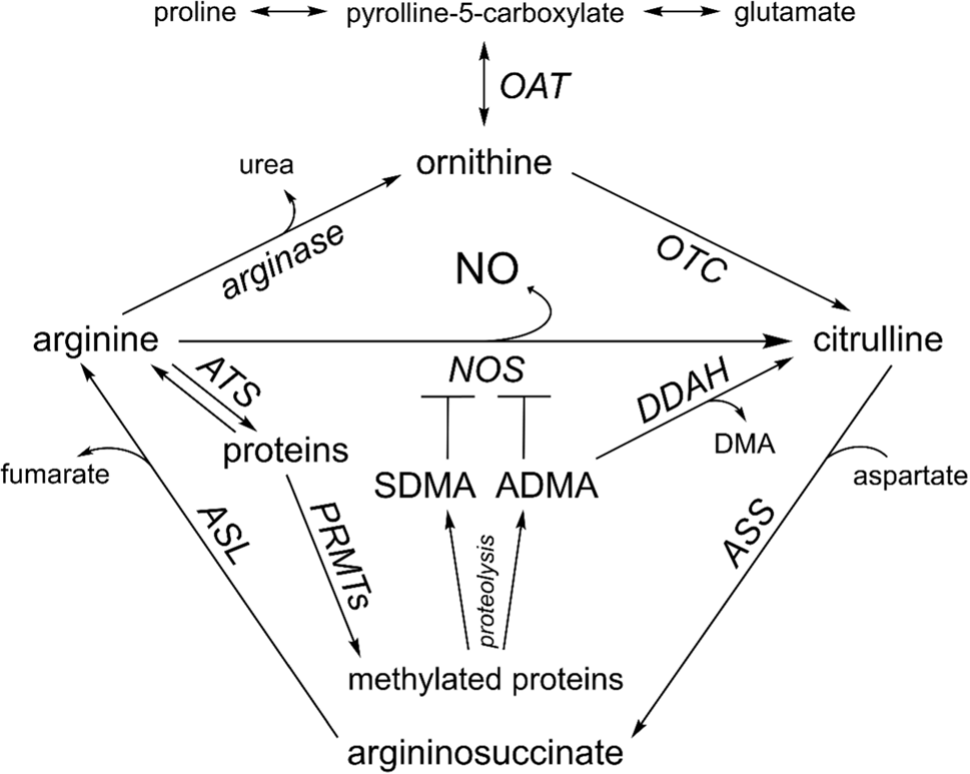
Biochemical pathways related to nitric oxide biosynthesis. *ADMA* asymmetric dimethylarginine, *ASL* argininosuccinate lyase, *ASS* argininosuccinate synthase, *ATS* arginyl-tRNA synthetase, *DDAH* dimethylarginine dimethylaminohydrolase, *DMA* dimethylamine, *NO* nitric oxide, *NOS* nitric oxide synthase, *OAT* ornithine aminotransferase, *OTC* ornithine transcarbamylase, *PRMTs* protein arginine *N*-methyltransferases, *SDMA* symmetric dimethylarginine

In the past, nitric oxide (NO) was considered a toxic gas associated with environmental pollution (Wu et al. 2018). However, it is now known that NO is one of the most potent physiological vasodilators that plays a very important role in the regulation of circulatory system (Li et al. 2009; Farah et al. 2018; Gantner et al. 2020). Endogenous NO is generated enzymatically by oxidation of l-arginine to l-citrulline (Fig. 1) in the presence of numerous cofactors. This reaction can be catalyzed by one of the three well-recognized isoforms of nitric oxide synthase (NOS): neuronal (nNOS), inducible (iNOS) or endothelial (eNOS) (Farah et al. 2018; Wu et al. 2018; Gheibi et al. 2020). eNOS occurring in vascular endothelial cells is the main source of nitric oxide under physiological conditions (Gkaliagkousi et al. 2009) and is characterized by constitutive activity (Moroz and Kohn 2011; Liu et al. 2018). Moreover, NO can be formed by non-enzymatic nitrate/nitrite reduction (Gheibi et al. 2020).

The bioavailability of l-arginine, as well as its metabolites, plays an essential role in the regulation of NO production. Methylation of arginine residues in proteins by protein arginine *N*-methyltransferases leads to the formation of asymmetric dimethylarginine (ADMA) and symmetric dimethylarginine (SDMA) (Böger 2003; Lin et al. 2016; Hannemann et al. 2021) (Fig. 1). Both ADMA and SDMA are inhibitors of NOS activity but have different inhibitory potential (Tsikas et al. 2018). SDMA is eliminated from the body by urinary excretion, while ADMA is removed mainly enzymatically and renal excretion is less important (Oliva-Damaso et al. 2019). As a result of enzymatic degradation of ADMA by dimethylarginine dimethylaminohydrolase (DDAH), citrulline and dimethylamine are formed (Böger 2003; Lin et al. 2016; Hannemann et al. 2021). In turn, l-arginine hydrolysis by the enzyme arginase leads to the formation of ornithine (Cederbaum et al. 2004), which can then be enzymatically converted into citrulline. Subsequently, citrulline can be reused to produce arginine via the argininosuccinate pathway (Lin et al. 2016) (Fig. 1).

Nitric oxide has been shown to play a cardio-protective role by regulating endothelium-dependent vasorelaxation via the soluble guanylyl cyclase (sGC)/cyclic guanosine monophosphate (cGMP)/protein kinase G (PKG) pathway (Ahmad et al. 2018; Ataei Ataabadi et al. 2020). Moreover, nitric oxide mediates angiogenesis, inhibits platelet aggregation and vascular smooth muscle cells proliferation (Gkaliagkousi et al. 2009; Farah et al. 2018). Furthermore, it has anti-adhesive properties—inhibits migration and adhesion of leukocytes and monocytes to the endothelium, and antioxidant properties—reduces production of superoxide radicals and oxidation of LDL cholesterol. Therefore, any reduction in physiological NO levels can lead to hypertension and/or atherosclerosis (Böger 2003; Gkaliagkousi et al. 2009). Additionally, nitric oxide controls renal hemodynamics and promotes diuresis and urinary excretion of sodium, as well as adaptation of kidneys to dietary salt intake (Gheibi et al. 2020). Therefore, it is postulated that renal NO production deficiency is the main mechanism for the development of systemic hypertension (Ahmad et al. 2018).

Hypertension and its complications are the leading causes of death and disability in aging societies. Spontaneously Hypertensive Rats (SHRs) are the most commonly used animal model to study hypertension. SHRs start to develop hypertension between the 4–6th week of age, and by 10 weeks their arterial blood pressure is 30% higher than in Wistar Kyoto (WKY) rats. 12-month-old SHRs showed characteristics of heart failure with preserved ejection fraction and compromised diastolic function (Huc et al. 2018). The SHR strain is derived from normotensive WKY rats (Okamoto and Aoki 1963).

The aim of this study was to evaluate the potential relationship between NO level in tissues collected from normotensive or hypertensive rats of different ages and the concentrations of NO-related endogenous metabolites in plasma and urine, which can provide a potential therapeutic approach to prevent the progression of hypertension via the NO pathway.

## Materials and methods

### Animals

The study assessed NO level in rat tissues and the concentration of NO-related pathway endogenous metabolites in rat plasma/urine. Biological material was collected from control rats in the study by Huc et al. (2018). The research was approved by the I Local Ethical Committee in Warsaw (permission no. 100/2016). The experiment was carried out on 16- and 60-week-old male normotensive Wistar Kyoto (WKY/Clzd) rats and age-matched male Spontaneously Hypertensive Rats (SHR/Clzd) from the Central Laboratory of Experimental Animals, Medical University of Warsaw (Warsaw, Poland). Animals were housed in groups (2–3 animals) in polypropylene cages, 12 h light/12 h dark cycle, temperature 22 °C, humidity 55%. Rats had access to food and water ad libitum. Four experimental groups were analyzed: 16-week-old WKY rats (16-WKY, *n* = 9), 60-week-old WKY rats (60-WKY, *n* = 7), 16-week-old SHRs (16-SHR, *n* = 7), and 60-week-old SHRs (60-SHR, *n* = 9).

### Blood and urine samples collection, tissue collection

The rats were housed for 2 days in metabolism cages to evaluate 24-h water balance and to collect urine for biochemical analysis. The following day, animals were anesthetized with an intraperitoneal injection of urethane (1.5 g/kg bw). After collecting blood from the right ventricle of the heart, the rats were killed by decapitation. Blood samples were collected in ethylenediaminetetraacetic acid (EDTA) tubes, and centrifuged for 15 min at 2500×*g* and 4 °C. During dissection, the hearts and kidneys were taken. Tissues, centrifuged plasma, and urine were immediately frozen in liquid nitrogen and kept at − 80 °C for further procedures.

### Nitric oxide level in tissue homogenates

To determine the total nitrate/nitrite concentration (a marker of nitric oxide level), rat hearts and kidneys were homogenized in ice-cold phosphate buffered saline pH 7.4 (1:5, w/v) for 1 min at 8000–9500 rpm using a blender homogenizer. The homogenates were centrifuged at 10,000×*g* for 20 min, and then the supernatants were ultra-filtered using 30 kDa molecular weight cut-off filters for 20 min. Total nitrate/nitrite concentration was determined using a colorimetric method according to the manufacturer’s instructions (780001, Cayman Chemicals, Ann Arbor, MI, United States). The nitric oxide level was expressed as µM of total NO_3_^−^/NO_2_^−^ concentration per 1 mg of protein.

### Protein level in tissue homogenates

To evaluate protein content, tissue samples were weighed and homogenized for 1 min at 8000–9500 rpm in ice-cold 0.1 M phosphate buffer pH 7.5 (1:4, w/v), then centrifuged for 10 min at 1600×*g*. Protein concentration in supernatants was determined by the method of Lowry et al. (1951) using crystalline BSA as a standard.

### Expression of eNOS gene

Total RNA was extracted from tissues using the Tri Reagent, according to the manufacturer’s protocol. Extracted RNA was suspended in nuclease-free water. Agarose gel electrophoresis and spectrophotometric measurement with NanoDrop ND-1000 (NanoDrop Technologies, Wilmington, DE, USA) were performed to assess the integrity and purity of the RNA samples. The RNA solutions were kept at − 80 °C until further procedures.

The total RNA from tissue samples was reverse-transcribed using the GoScript™ Reverse Transcriptase Kit according to the manufacturer’s instructions (Promega, Madison, WI, USA). The detailed mix composition and reaction conditions were described in the previous study (Szlęzak et al. 2021). The cDNA solutions were kept at − 20 °C.

eNOS gene expression was determined by quantitative real-time PCR (qPCR) analysis using the MiniOpticon™ System (Bio-Rad, Hercules, CA, USA), Fast SYBR Green qPCR Master Mix (EURx, Gdańsk, Poland), and specific primers (Sigma-Aldrich, Darmstadt, Germany). qPCR amplifications consisting of 40 cycles of denaturation and annealing/extension were performed according to the protocol provided by the manufacturer. EEF1A1 (eukaryotic translation elongation factor 1 alpha 1) gene was used as a reference gene. The primer sequences were as follows: eNOS forward 5′-TAACTAGACTGGGAGGGAGTCA-3′ and eNOS reverse 5′-AAAGCATACGAAGAGGGCAG-3′ (product size 88 bp), EEF1A1 forward 5′-TTTCGCAACGGGTTTGCC-3′ and EEF1A1 reverse 5′-GCCGGAATCTACGTGTCCAA-3′ (product size 121 bp) (Szlęzak et al. 2022). eNOS primers were designed using the NCBI OMIM database and NCBI Primer-BLAST tool. The mRNA expression levels of target gene were calculated by the 2^−ΔCq^ method.

### Level of NO-related pathway endogenous metabolites and dimethylarginines

The UPLC-MS/MS (ultra-performance liquid chromatography–tandem mass spectrometry) method was used to determine plasma and urine concentrations of arginine, ornithine, citrulline, and asymmetric and symmetric dimethylarginine. The analysis was performed by a Waters Acquity ultra-performance liquid chromatograph coupled with a Waters TQ-S triple-quadrupole mass spectrometer. Separations were carried out with a Waters HILIC column (1.7 μm, 2.1 mm × 50 mm). A mobile phase consisting of solvent A—water containing 0.1% ammonium hydroxide and solvent B—acetonitrile containing 0.1% formic acid was used to elute the samples. Multiple reaction monitoring (MRM) via positive electrospray ionization (ESI +) mode on a mass spectrometer was used. Samples were prepared using acetone—10 μl of the sample was mixed with 100 μl of acetone containing internal standards (isotope-labeled ADMA-D_6_, arginine-D_4_ and ornithine-^13^C_5_), the injection volume was 7 μl (Gąsecka et al. 2021). The internal standard at known concentration was added to each sample (calibrators, plasma, and urine) to determine the response which is used to generate a calibration curve and quantify the concentration of the analytes. The calibration curve was generated by comparing a ratio of the analyte’s peak area to the peak of the internal standard against known analyte concentration. Biological samples were compared with an obtained calibration curve and concentrations were calculated. The analyses were carried out at the Mass Spectrometry Laboratory of the Institute of Biochemistry and Biophysics Polish Academy of Sciences (Warsaw, Poland).

### Statistical analysis

All results were represented as arithmetic means with standard deviations (SD). The lack of normal distribution of the data and the lack of homogeneity of variance were confirmed by the Shapiro–Wilk test and the Levene test, respectively. Statistical analysis was performed via the Mann–Whitney *U* test. Differences with a *p* value < 0.05 were considered statistically significant. Analyses were conducted using Statsoft Statistica 13 software (Tibco, Palo Alto, CA, USA).

## Results

### Animal parameters

Detailed metabolic and hemodynamic rat parameters were previously described in the study by Huc et al. (2018). The following mean arterial blood pressure values ± SE were observed in anesthetized WKY rats: 83.73 ± 4.46 mmHg in 16-week-old and 81.79 ± 2.19 mmHg in 60-week-old WKY. Significantly higher values (*p* < 0.05 vs. age-matched WKY groups) were observed in anesthetized SHR rats: 101.6 ± 4.34 mmHg in 16-week-old and 120.98 ± 5.03 mmHg in 60-week-old SHRs. In the 16-week-old animal groups, higher food intake and stool output, and lower water intake were observed in WKY rats compared to SHRs. The body mass of these rats was similar. There were no differences in food and water intake, stool output, and body mass in the groups of 60-week-old rats. The mean 24-h urine output values ± SE were as follows: 10.14 ± 0.57 ml in 16-week-old WKY, 15.87 ± 2.33 ml in 60-week-old WKY, 15.14 ± 1.36 ml in 16-week-old SHR and 16.16 ± 1.66 ml in 60-week-old SHR (Huc et al. 2018).

### NO level in tissues

**Fig. 2:**
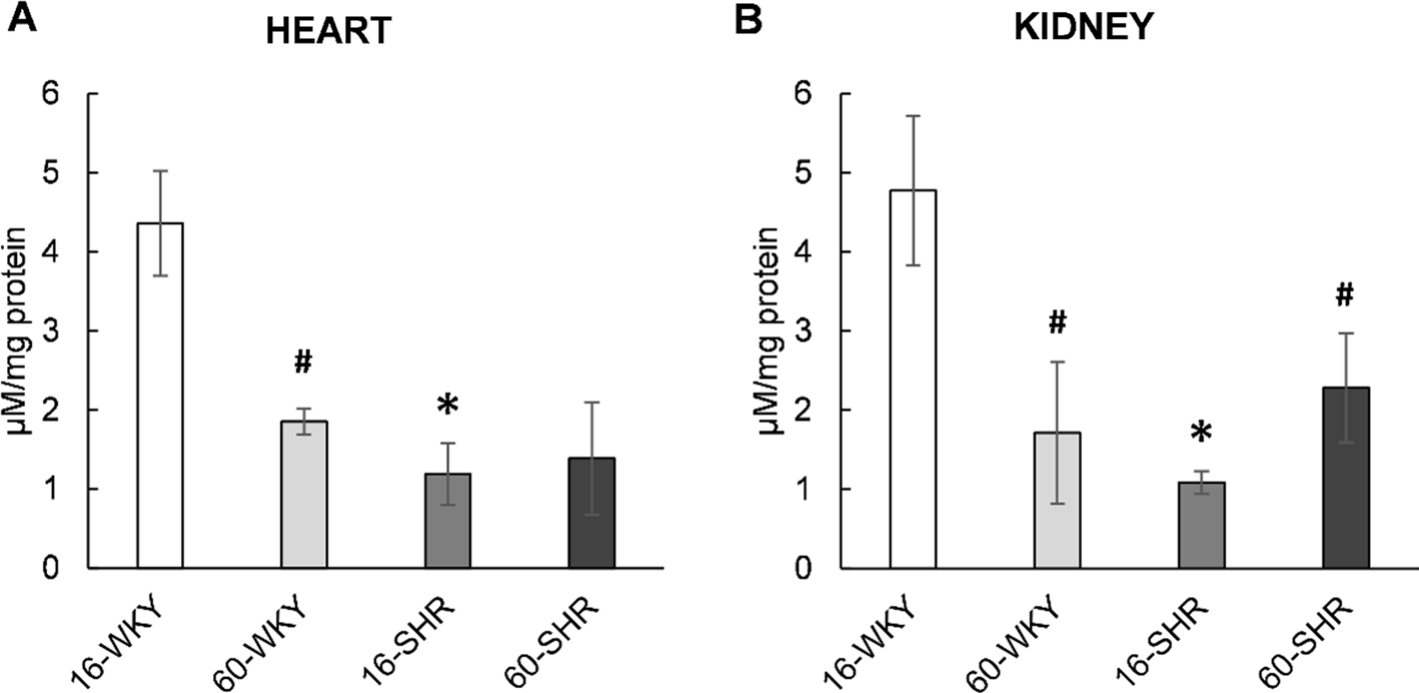
Total nitrate/nitrite concentrations in the hearts (**A**) and kidneys (**B**) of rats. Values represent an arithmetic mean ± SD of 6–12 repetitions for 2–4 animals per group. *16-WKY* 16-week-old WKY rats, *60-WKY* 60-week-old WKY rats, *16-SHR* 16-week-old SHRs, *60-SHR* 60-week-old SHRs. *p < 0.05 SHR vs. WKY of the same age, ^#^*p* < 0.05 60-week-old vs. 16-week-old

Total nitrate/nitrite concentrations in tissue homogenates were used to determine nitric oxide levels in the hearts and kidneys of rats (Fig. 2). The highest levels of nitrate/nitrite were observed in the hearts (Fig. 2A) and kidneys (Fig. 2B) of 16-week-old WKY rats, and these values were more than three times higher compared to tissues of age-matched SHRs and more than two times higher compared to tissues of 60-week-old WKY rats. In hypertensive rats, NO level in the hearts was the same in both age groups, while in the kidneys it was statistically significantly higher in 60-week-old SHRs compared to 16-week-old SHRs.

### eNOS gene expression at the mRNA level

**Fig. 3:**
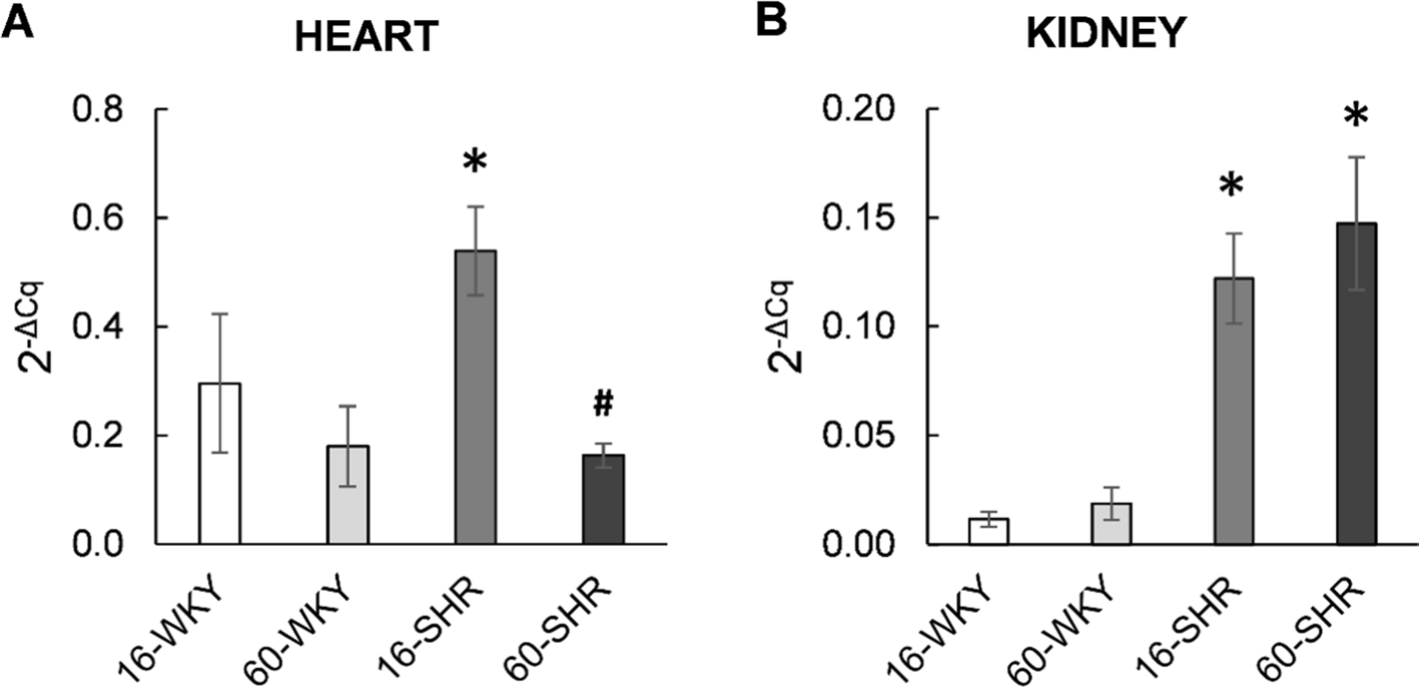
Expression of the eNOS gene in the hearts (**A**) and kidneys (**B**) of rats. Values represent an arithmetic mean ± SD (*n* = 3 animals per group). Each value is the mean of at least two independent experiments. *16-WKY* 16-week-old WKY rats, *60-WKY* 60-week-old WKY rats, *16-SHR* 16-week-old SHRs, *60-SHR* 60-week-old SHRs. *p < 0.05 SHR vs. WKY of the same age, ^#^*p* < 0.05 60-week-old vs. 16-week-old

The highest level of eNOS gene expression was found in the hearts of 16-week-old hypertensive rats and this value was significantly higher than in the 60-week-old SHRs and 16-week-old WKY rats (Fig. 3A). No statistically significant changes were observed in the expression of the endothelial NOS gene in groups of normotensive rats of different age, but there was a tendency to decrease this expression in the hearts of 60-week-old animals. The expression of the eNOS gene in the kidneys of WKY rats in both age groups was low (Fig. 3B), a significantly higher expression level was found in the groups of rats with hypertension.

### Plasma concentrations and urinary excretion of NO-related pathway endogenous metabolites

**Fig. 4:**
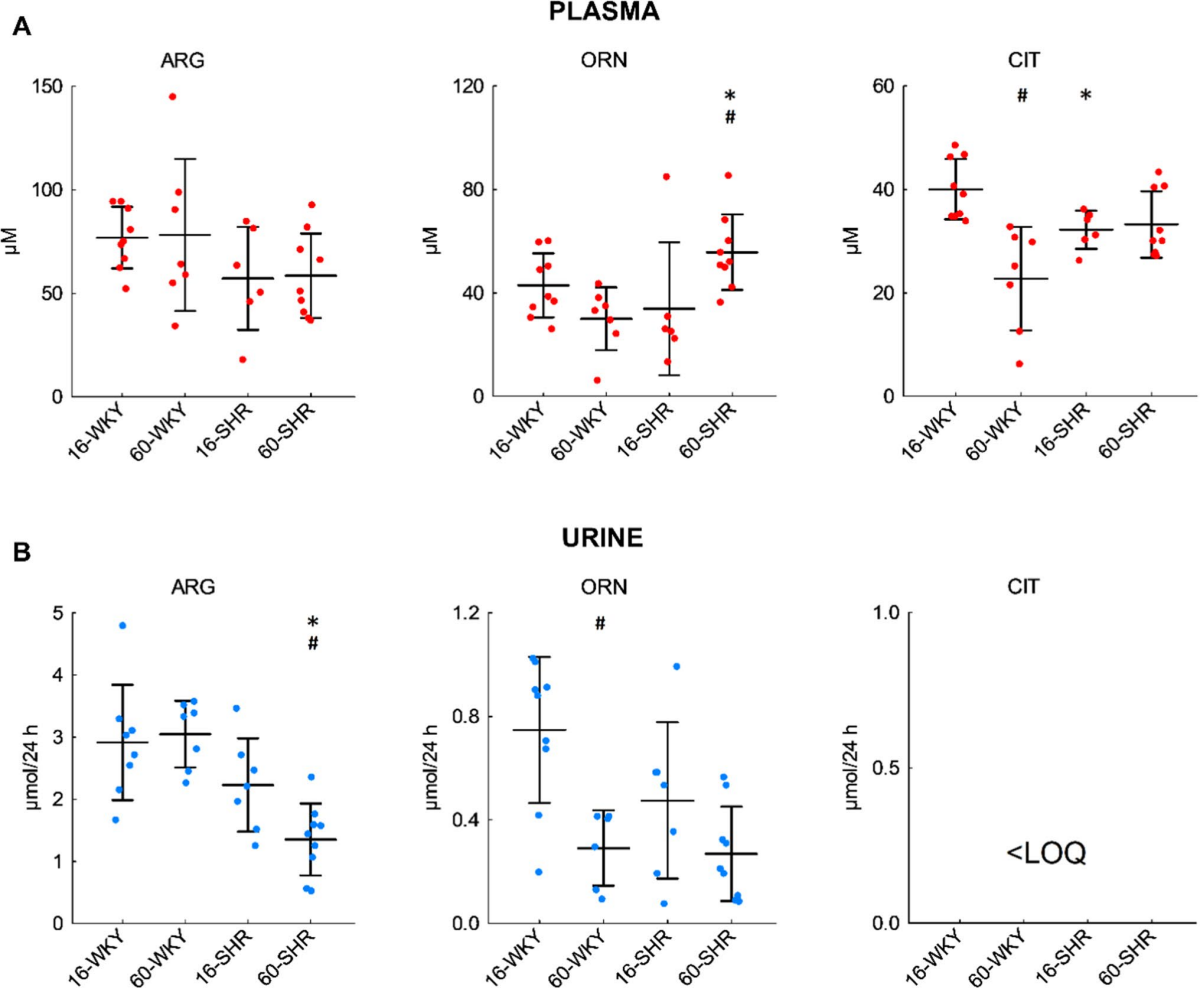
Plasma concentrations (**A**) and urinary excretion (**B**) of NO-related pathway endogenous metabolites in rats. The concentrations in the urine samples were normalized to 24-h urinary excretion. Values represent an arithmetic mean ± SD (*n* = 6–9 animals per group). The colored circles show the individual data values. *16-WKY* 16-week-old WKY rats, *60-WKY* 60-week-old WKY rats, *16-SHR* 16-week-old SHRs, *60-SHR* 60-week-old SHRs, *ARG* arginine, *CIT* citrulline, *ORN* ornithine, *< LOQ* below the limit of quantification. The quantification limits were 4.03 µM for arginine, 5.04 µM for ornithine, and 5.85 µM for citrulline. *p < 0.05 SHR vs. WKY of the same age, ^#^*p* < 0.05 60-week-old vs. 16-week-old

There were no statistically significant differences in the plasma arginine concentration of the examined groups of rats (Fig. 4A). Plasma ornithine level was significantly higher in older hypertensive rats than in age-matched WKY rats and 16-week-old SHRs. In turn, concentration of citrulline was significantly higher in the plasma of 16-week-old WKY rats compared to 60-week-old WKY and 16-week-old SHRs.

The amount of arginine excreted in urine during 24 h was lowest in the group of 60-week-old hypertensive rats (Fig. 4B). 16-week-old WKY rats excreted the most ornithine and this value was significantly higher compared to older WKY rats. No citrulline was detected in rats’ urine.

In the studied groups, no statistically significant correlation was found between plasma concentrations and urinary excretion of arginine and ornithine (data not shown).

### Plasma concentrations and urinary excretion of asymmetric and symmetric dimethylarginine

**Fig. 5:**
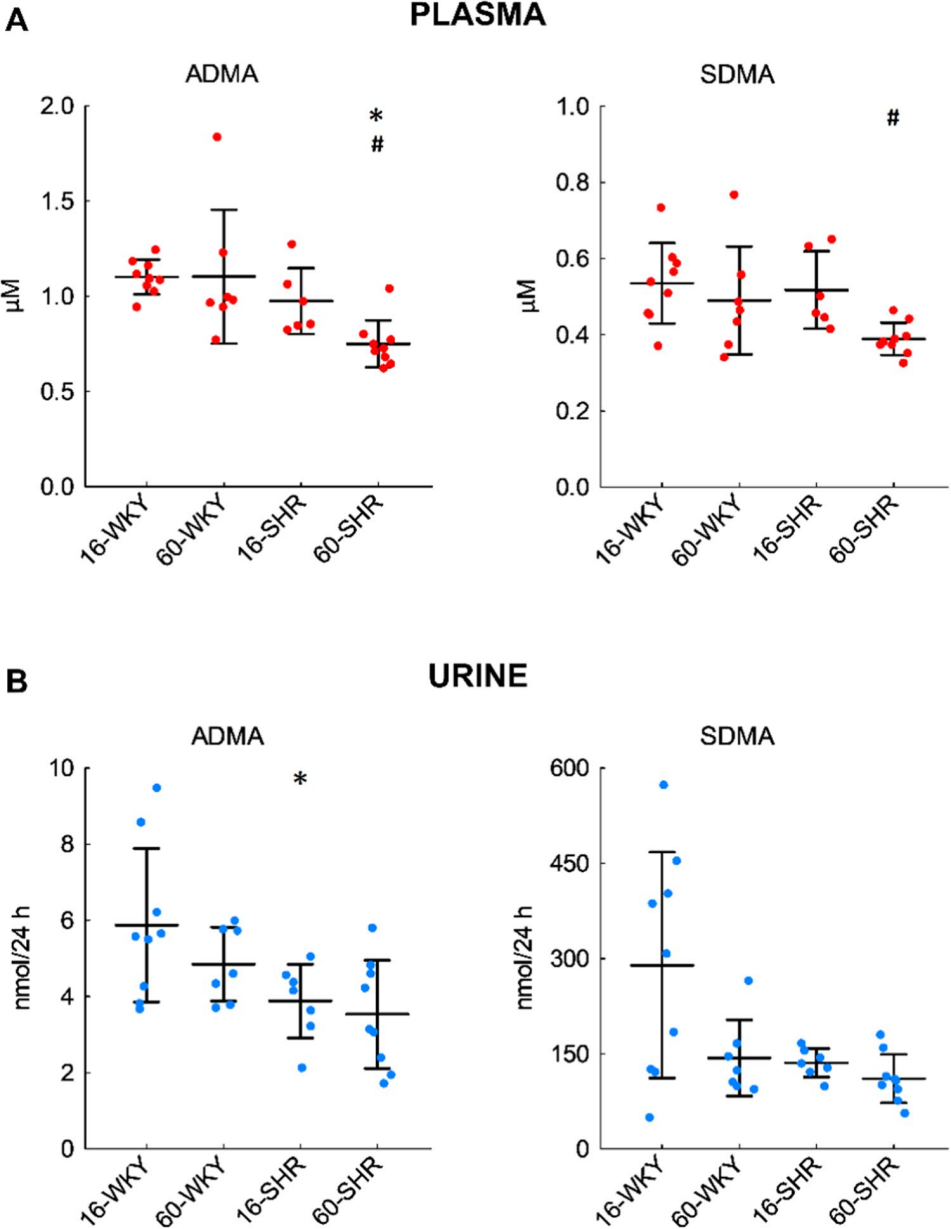
Plasma concentrations (**A**) and urinary excretion (**B**) of ADMA and SDMA in rats. The concentrations in the urine samples were normalized to 24-h urinary excretion. Values represent an arithmetic mean ± SD (*n* = 6–9 animals per group). The colored circles show the individual data values. *16-WKY* 16-week-old WKY rats, *60-WKY* 60-week-old WKY rats, *16-SHR* 16-week-old SHRs, *60-SHR* 60-week-old SHRs, *ADMA* asymmetric dimethylarginine, *SDMA* symmetric dimethylarginine. The quantification limits were 0.03 µM for ADMA and 0.02 µM for SDMA. *p < 0.05 SHR vs. WKY of the same age, ^#^*p* < 0.05 60-week-old vs. 16-week-old

The concentrations of ADMA in plasma were similar in all groups of rats (Fig. 5A), except for the 60-week-old SHRs, which showed statistically lower levels of ADMA than age-matched WKY rats and 16-week-old SHRs. Mean plasma SDMA concentrations in normotensive and 16-week-old hypertensive rats ranged from 0.49 to 0.54 µM (Fig. 5A). In the plasma of 60-week-old hypertensive rats, a statistically significant decrease in SDMA level was observed compared to 16-week-old SHRs.

The mean 24-h urinary excretion of ADMA and SDMA was the highest in the group of 16-week-old WKY rats (Fig. 5B). However, statistically significant differences were observed only in the amount of ADMA in the urine of 16-week-old SHR compared to age-matched WKY rats.

## Discussion

Our research shows that hypertension and aging decrease tissue NO levels and reduce urinary excretion of NOS inhibitors, i.e., ADMA and SDMA, in rats. Furthermore, it provides original data on changes in the expression of the eNOS gene at the mRNA level in rat tissues depending on age and absence/presence of hypertension.

The experiments confirmed that NO level undergoes a reduction in heart and kidney homogenates from hypertensive and 60-week-old normotensive rats. These changes occurred independently of changes in eNOS gene expression. In the cardiovascular system, all three NOS isoforms have regulatory functions. nNOS is responsible for central regulation of blood pressure, iNOS plays a protective role in septic shock, and eNOS determines vasodilation, vasoprotection, and prevention of atherosclerosis (Förstermann and Sessa 2012). The crucial role of eNOS in the pathogenesis of hypertension has been confirmed by the observation that eNOS-knockout mice develop hypertension (Huang et al. 1995; Shesely et al. 1996). Nevertheless, there is a lack of research on the level of eNOS gene expression in tissues of normotensive and hypertensive rats, and reports on the effect of age on this expression in rats’ vessels are contradictory (El Assar et al. 2013). In our study, the expression of eNOS in SHRs tissues was (depending on the type of tissue and the age of the animals) higher or similar to that in WKY rats. Previous studies using human and bovine aortic endothelial cells showed that eNOS expression increases at the mRNA level under the influence of reactive oxygen species (ROS) such as hydrogen peroxide (H_2_O_2_) (Drummond et al. 2000). ROS are important modulators in cardiovascular diseases and hypertension (Davalli et al. 2016)—their increased levels in the myocardium in SHRs compared to WKY rats have been experimentally demonstrated (Shi et al. 2007). However, the level of eNOS gene expression does not necessarily translate into the amount of nitric oxide formed, since the activity of NOS is regulated by many cofactors. The shortage of cofactors leads to eNOS uncoupling and the formation of superoxide anions (Münzel et al. 2005), which in turn react with NO and generate highly reactive peroxynitrite (ONOO^–^) associated with age-related vascular dysfunction (El Assar et al. 2013). Nitric oxide deficiency is associated with increased blood pressure, as well as with cardiovascular and kidney diseases (Lin et al. 2016). Decreased endogenous NO concentrations have been reported in the plasma of patients with essential hypertension (Node et al. 1997) and in the urine of patients with chronic kidney disease (Schmidt and Baylis 2000).

The levels of metabolites, such as arginine, citrulline, and ornithine, are closely related to the maintenance of NO homeostasis (Fig. 1). The important source of arginine for nitric oxide synthesis in endothelial cells of blood vessels is extracellular arginine, present in plasma (Wu and Meininger 2002). In our experiment, no statistically significant changes were observed in mean plasma arginine concentrations of hypertensive rats compared to normotensive rats, although there was a trend toward a decrease in mean arginine concentrations in SHRs. The high level of citrulline observed in the plasma of 16-week-old normotensive rats was correlated with the high level of nitric oxide in the tissues of these rats. In turn, different plasma concentrations of ornithine could have been caused by the differential activity of arginase, considered to be the key regulator of NO availability, which influences NOS activity using their common substrate (Rabelo et al. 2015). White et al. (2006) showed that the aortic NO production of 3-month-old WKY rats is higher compared to 23-month-old rats with simultaneous higher NOS activity and decreased arginase activity. In turn, skeletal muscle arterioles isolated from salt-loaded hypertensive Dahl rats showed higher levels of arginase compared to low-salt normotensive controls (Johnson et al. 2005). Similarly, it was found that in diabetic hypertensive rats the activity of serum arginase was increased, and inhibition of this enzyme was associated with a reduction in diastolic blood pressure and an increased aortic NO production (El-Bassossy et al. 2012). Kondziella et al. (2008) showed that plasma levels of ornithine were higher in SHRs compared to WKY control rats. In our study, we found a significantly higher ornithine concentration in the plasma of 60-week-old hypertensive rats, which may be a determinant of arginase activity.

Previous papers have shown that increased plasma ADMA levels are associated with diseases related to endothelial dysfunction, such as cardiovascular diseases, diabetes (Tain and Hsu 2017), as well as with chronic kidney disease (Baylis et al. 2012). It is known that ADMA contributes to the development of hypertension in two ways: by inhibiting the activity of eNOS, which causes vasoconstriction, and by reducing renal NO synthesis, which may lead to suppression of sodium excretion by the kidneys (Oliva-Damaso et al. 2019). Achan et al. (2003) showed that intravenous administration of low doses of ADMA leads to the development of hypertension and cardiac dysfunction in humans. Increased plasma ADMA concentrations are considered a risk factor for premature mortality in renal transplant recipients (Frenay et al. 2015). On the other hand, high plasma SDMA levels have been associated with hypertension and hyperlipidemia in patients with rheumatoid arthritis (Chandrasekharan et al. 2018). Also, the level of urinary ADMA and/or SDMA seems to be an important parameter in the assessment of the patient's condition. The study by Said et al. (2019) showed that high urinary excretion of ADMA and SDMA in renal transplant recipients was associated with a lower risk of cardiovascular diseases mortality. Similarly, in patients with coronary artery disease, low urinary ADMA concentrations have been associated with impaired heart function and increased mortality (Wolf et al. 2012). In our experiment, 16-week-old normotensive rats excreted the most ADMA and SDMA. However, in all experimental groups, the average amount of symmetric dimethylarginine excreted in the urine was significantly higher (29–49 times on average) than the average amount of asymmetric dimethylarginine. Previously, a comparable effect was observed in male Sprague Dawley rats, where the amount of excreted SDMA was on average 22–25 times higher than ADMA (Al Banchaabouchi et al. 2000; Matsuguma et al. 2006).

**Fig. 6:**
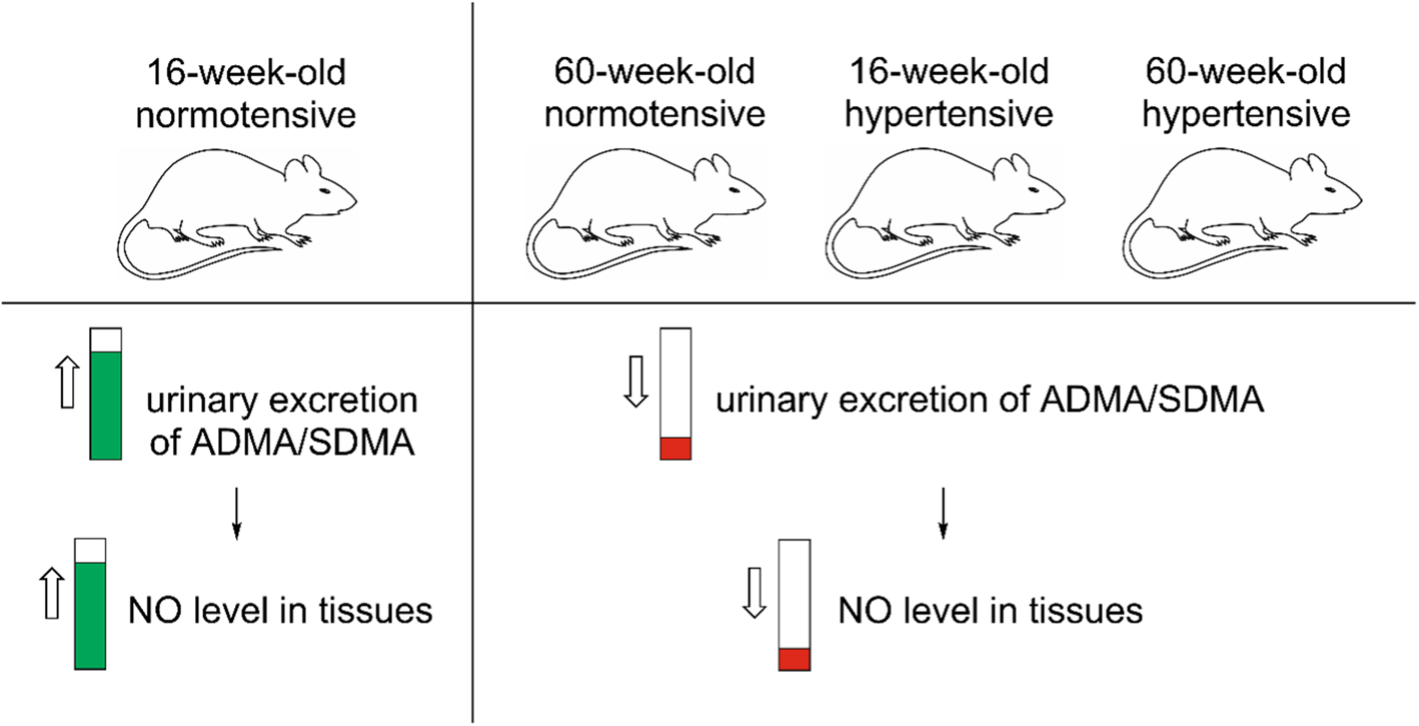
Proposed dependence between urinary excretion of dimethylarginines and NO level in tissues of normotensive/hypertensive rats of different ages. *ADMA* asymmetric dimethylarginine, *NO* nitric oxide, *SDMA* symmetric dimethylarginine

This study has shown that 16-week-old WKY rats were characterized by the highest total concentration of nitrate/nitrite in tissue homogenates. At the same time, there was the highest 24-h urinary excretion of ADMA and SDMA in the group of these rats. On this basis, we suggest that urinary elimination of these dimethylarginines is positively related to NO level in rat tissues (Fig. 6). However, plasma concentrations of arginine, ADMA, and SDMA were comparable in all study groups. Therefore, we hypothesize that tissue protein turnover plays a key role in NO formation and ADMA/SDMA homeostasis.

In addition to the above, there is some evidence of a crosstalk between nitric oxide and hydrogen sulfide (H_2_S). Hosoki et al. (1997) showed that the simultaneous use of H_2_S and NO donors induced greater relaxation of vascular smooth muscle than one or the other molecule alone. On the other hand, hydrogen sulfide donors protected endothelial NO production under oxidative stress conditions (Al-Magableh et al. 2014). Studies in mice have shown that hydrogen sulfide therapy protects against heart failure (Kondo et al. 2013) and attenuates I/R injury (King et al. 2014) by increasing eNOS activity. In turn, Zhao et al. (2001) showed that incubation of homogenized rat vascular tissues with different doses of NO donor enhances the production of H_2_S in a concentration-dependent manner. Our current and previous examination (Szlęzak et al. 2022) indicates a correlation between the formation of nitric oxide and hydrogen sulfide in the examined tissues—their levels were reduced in the hearts and kidneys of 60-week-old normotensive rats and hypertensive rats in both age groups. The obtained results confirm the significant relationship between these two molecules under physiological conditions and provide the basis for further, more advanced research.

The limitation of the presented studies is that the individual methods used (total nitrate/nitrite determination, protein content determination) require separate material, a different tissue fragment, and different homogenate preparation procedures, which, however, should not significantly affect the final results.

## Conclusions

In summary, our study shows that NO level is reduced in the hearts and kidneys of hypertensive and older normotensive rats, however, this is not dependent on eNOS gene expression. Increased urinary excretion of ADMA and SDMA in younger normotensive rats may suggest that higher elimination of ADMA and SDMA from the body increases the level of nitric oxide in rat tissues (Fig. 6). Further studies evaluating the effects of ADMA and SDMA are needed to understand the role of the methylamines in hypertension and aging-related processes.

## Data Availability

The authors confirm that the data supporting the findings of this study are included in the article.

## References

[CR1] Achan V, Broadhead M, Malaki M, Whitley G, Leiper J, MacAllister R, Vallance P (2003). Asymmetric dimethylarginine causes hypertension and cardiac dysfunction in humans and is actively metabolized by dimethylarginine dimethylaminohydrolase. Arterioscler Thromb Vasc Biol.

[CR2] Ahmad A, Dempsey SK, Daneva Z, Azam M, Li N, Li PL, Ritter JK (2018). Role of nitric oxide in the cardiovascular and renal systems. Int J Mol Sci.

[CR3] Al Banchaabouchi M, Marescau B, Possemiers I, D'Hooge R, Levillain O, De Deyn PP (2000). NG, NG-dimethylarginine and NG, NG-dimethylarginine in renal insufficiency. Pflugers Arch.

[CR4] Al-Magableh MR, Kemp-Harper BK, Ng HH, Miller AA, Hart JL (2014). Hydrogen sulfide protects endothelial nitric oxide function under conditions of acute oxidative stress in vitro. Naunyn Schmiedebergs Arch Pharmacol.

[CR5] Ataei Ataabadi E, Golshiri K, Jüttner A, Krenning G, Danser AHJ, Roks AJM (2020). Nitric oxide-cGMP signaling in hypertension: current and future options for pharmacotherapy. Hypertension.

[CR6] Baylis C (2012). Nitric oxide synthase derangements and hypertension in kidney disease. Curr Opin Nephrol Hypertens.

[CR7] Böger RH (2003). The emerging role of asymmetric dimethylarginine as a novel cardiovascular risk factor. Cardiovasc Res.

[CR8] Cederbaum SD, Yu H, Grody WW, Kern RM, Yoo P, Iyer RK (2004). Arginases I and II: do their functions overlap?. Mol Genet Metab.

[CR9] Chandrasekharan UM, Wang Z, Wu Y, Wilson Tang WH, Hazen SL, Wang S, Elaine Husni M (2018). Elevated levels of plasma symmetric dimethylarginine and increased arginase activity as potential indicators of cardiovascular comorbidity in rheumatoid arthritis. Arthritis Res Ther.

[CR10] Davalli P, Mitic T, Caporali A, Lauriola A, D'Arca D (2016). ROS, cell senescence, and novel molecular mechanisms in aging and age-related diseases. Oxid Med Cell Longev.

[CR11] Drummond GR, Cai H, Davis ME, Ramasamy S, Harrison DG (2000). Transcriptional and posttranscriptional regulation of endothelial nitric oxide synthase expression by hydrogen peroxide. Circ Res.

[CR12] El Assar M, Angulo J, Rodríguez-Mañas L (2013). Oxidative stress and vascular inflammation in aging. Free Radic Biol Med.

[CR13] El-Bassossy HM, El-Fawal R, Fahmy A (2012). Arginase inhibition alleviates hypertension associated with diabetes: effect on endothelial dependent relaxation and NO production. Vascul Pharmacol.

[CR14] Farah C, Michel LYM, Balligand JL (2018). Nitric oxide signalling in cardiovascular health and disease. Nat Rev Cardiol.

[CR15] Förstermann U, Sessa WC (2012). Nitric oxide synthases: regulation and function. Eur Heart J.

[CR16] Frenay AR, van den Berg E, de Borst MH, Beckmann B, Tsikas D, Feelisch M, Navis G, Bakker SJ, van Goor H (2015). Plasma ADMA associates with all-cause mortality in renal transplant recipients. Amino Acids.

[CR17] Gantner BN, LaFond KM, Bonini MG (2020). Nitric oxide in cellular adaptation and disease. Redox Biol.

[CR18] Gąsecka A, Szwed P, Jasińska K, Fidali O, Kłębukowska A, Eyileten C, Postula M, Szarpak Ł, Mazurek T, Opolski G, Filipiak KJ, Ufnal M (2021). Symmetric dimethylarginine is altered in patients after myocardial infarction and predicts adverse outcomes. J Inflamm Res.

[CR19] Gheibi S, Samsonov AP, Gheibi S, Vazquez AB, Kashfi K (2020). Regulation of carbohydrate metabolism by nitric oxide and hydrogen sulfide: Implications in diabetes. Biochem Pharmacol.

[CR20] Gkaliagkousi E, Douma S, Zamboulis C, Ferro A (2009). Nitric oxide dysfunction in vascular endothelium and platelets: role in essential hypertension. J Hypertens.

[CR21] Hannemann J, Siques P, Schmidt-Hutten L, Zummack J, Brito J, Böger R (2021). Association of genes of the NO pathway with altitude disease and hypoxic pulmonary hypertension. J Clin Med.

[CR22] Hosoki R, Matsuki N, Kimura H (1997). The possible role of hydrogen sulfide as an endogenous smooth muscle relaxant in synergy with nitric oxide. Biochem Biophys Res Commun.

[CR23] Huang PL, Huang Z, Mashimo H, Bloch KD, Moskowitz MA, Bevan JA, Fishman MC (1995). Hypertension in mice lacking the gene for endothelial nitric oxide synthase. Nature.

[CR24] Huc T, Drapala A, Gawrys M, Konop M, Bielinska K, Zaorska E, Samborowska E, Wyczalkowska-Tomasik A, Pączek L, Dadlez M, Ufnal M (2018). Chronic, low-dose TMAO treatment reduces diastolic dysfunction and heart fibrosis in hypertensive rats. Am J Physiol Heart Circ Physiol.

[CR25] Johnson FK, Johnson RA, Peyton KJ, Durante W (2005). Arginase inhibition restores arteriolar endothelial function in Dahl rats with salt-induced hypertension. Am J Physiol Regul Integr Comp Physiol.

[CR26] King AL, Polhemus DJ, Bhushan S, Otsuka H, Kondo K, Nicholson CK, Bradley JM, Islam KN, Calvert JW, Tao YX, Dugas TR, Kelley EE, Elrod JW, Huang PL, Wang R, Lefer DJ (2014). Hydrogen sulfide cytoprotective signaling is endothelial nitric oxide synthase-nitric oxide dependent. Proc Natl Acad Sci U S A.

[CR27] Kondo K, Bhushan S, King AL, Prabhu SD, Hamid T, Koenig S, Murohara T, Predmore BL, Gojon G, Gojon G, Wang R, Karusula N, Nicholson CK, Calvert JW, Lefer DJ (2013). H_2_S protects against pressure overload-induced heart failure via upregulation of endothelial nitric oxide synthase. Circulation.

[CR28] Kondziella D, Zetterberg H, Haugen E, Fu M (2008). Hypertension in spontaneously hypertensive rats occurs despite low plasma levels of homocysteine. Physiol Res.

[CR29] Li L, Hsu A, Moore PK (2009). Actions and interactions of nitric oxide, carbon monoxide and hydrogen sulphide in the cardiovascular system and in inflammation—a tale of three gases!. Pharmacol Ther.

[CR30] Lin IC, Hsu CN, Lo MH, Chien SJ, Tain YL (2016). Low urinary citrulline/arginine ratio associated with blood pressure abnormalities and arterial stiffness in childhood chronic kidney disease. J Am Soc Hypertens.

[CR31] Liu X, Xu X, Shang R, Chen Y (2018). Asymmetric dimethylarginine (ADMA) as an important risk factor for the increased cardiovascular diseases and heart failure in chronic kidney disease. Nitric Oxide.

[CR32] Lowry OH, Rosebrough NJ, Farr AL, Randall RJ (1951). Protein measurement with the Folin phenol reagent. J Biol Chem.

[CR33] Matsuguma K, Ueda S, Yamagishi S, Matsumoto Y, Kaneyuki U, Shibata R, Fujimura T, Matsuoka H, Kimoto M, Kato S, Imaizumi T, Okuda S (2006). Molecular mechanism for elevation of asymmetric dimethylarginine and its role for hypertension in chronic kidney disease. J Am Soc Nephrol.

[CR34] Moroz LL, Kohn AB (2011). Parallel evolution of nitric oxide signaling: diversity of synthesis and memory pathways. Front Biosci (landmark Ed).

[CR35] Münzel T, Daiber A, Ullrich V, Mülsch A (2005). Vascular consequences of endothelial nitric oxide synthase uncoupling for the activity and expression of the soluble guanylyl cyclase and the cGMP-dependent protein kinase. Arterioscler Thromb Vasc Biol.

[CR36] Node K, Kitakaze M, Yoshikawa H, Kosaka H, Hori M (1997). Reduced plasma concentrations of nitrogen oxide in individuals with essential hypertension. Hypertension.

[CR37] Okamoto K, Aoki K (1963). Development of a strain of spontaneously hypertensive rats. Jpn Circ J.

[CR38] Oliva-Damaso E, Oliva-Damaso N, Rodriguez-Esparragon F, Payan J, Baamonde-Laborda E, Gonzalez-Cabrera F, Santana-Estupiñan R, Rodriguez-Perez JC (2019). Asymmetric (ADMA) and symmetric (SDMA) dimethylarginines in chronic kidney disease: a clinical approach. Int J Mol Sci.

[CR39] Rabelo LA, Ferreira FO, Nunes-Souza V, da Fonseca LJ, Goulart MO (2015). Arginase as a critical prooxidant mediator in the binomial endothelial dysfunction-atherosclerosis. Oxid Med Cell Longev.

[CR40] Said MY, Bollenbach A, Minović I, van Londen M, Frenay AR, de Borst MH, van den Berg E, Kayacelebi AA, Tsikas D, van Goor H, Navis G, Bakker SJL (2019). Plasma ADMA, urinary ADMA excretion, and late mortality in renal transplant recipients. Amino Acids.

[CR41] Schmidt RJ, Baylis C (2000). Total nitric oxide production is low in patients with chronic renal disease. Kidney Int.

[CR42] Shesely EG, Maeda N, Kim HS, Desai KM, Krege JH, Laubach VE, Sherman PA, Sessa WC, Smithies O (1996). Elevated blood pressures in mice lacking endothelial nitric oxide synthase. Proc Natl Acad Sci U S A.

[CR43] Shi YX, Chen Y, Zhu YZ, Huang GY, Moore PK, Huang SH, Yao T, Zhu YC (2007). Chronic sodium hydrosulfide treatment decreases medial thickening of intramyocardial coronary arterioles, interstitial fibrosis, and ROS production in spontaneously hypertensive rats. Am J Physiol Heart Circ Physiol.

[CR44] Szlęzak D, Bronowicka-Adamska P, Hutsch T, Ufnal M, Wróbel M (2021). Hypertension and aging affect liver sulfur metabolism in rats. Cells.

[CR45] Szlęzak D, Hutsch T, Ufnal M, Wróbel M (2022). Heart and kidney H_2_S production is reduced in hypertensive and older rats. Biochimie.

[CR46] Tain YL, Hsu CN (2017). Toxic dimethylarginines: asymmetric dimethylarginine (ADMA) and symmetric dimethylarginine (SDMA). Toxins (basel).

[CR47] Tsikas D, Bollenbach A, Hanff E, Kayacelebi AA (2018). Asymmetric dimethylarginine (ADMA), symmetric dimethylarginine (SDMA) and homoarginine (hArg): the ADMA, SDMA and hArg paradoxes. Cardiovasc Diabetol.

[CR48] White AR, Ryoo S, Li D, Champion HC, Steppan J, Wang D, Nyhan D, Shoukas AA, Hare JM, Berkowitz DE (2006). Knockdown of arginase I restores NO signaling in the vasculature of old rats. Hypertension.

[CR49] Wolf C, Lorenzen JM, Stein S, Tsikas D, Störk S, Weidemann F, Ertl G, Anker SD, Bauersachs J, Thum T (2012). Urinary asymmetric dimethylarginine (ADMA) is a predictor of mortality risk in patients with coronary artery disease. Int J Cardiol.

[CR50] Wu G, Meininger CJ (2002). Regulation of nitric oxide synthesis by dietary factors. Annu Rev Nutr.

[CR51] Wu D, Hu Q, Zhu D (2018). An update on hydrogen sulfide and nitric oxide interactions in the cardiovascular system. Oxid Med Cell Longev.

[CR52] Zhao W, Zhang J, Lu Y, Wang R (2001). The vasorelaxant effect of H(2)S as a novel endogenous gaseous K(ATP) channel opener. EMBO J.

